# The efficacy and safety of Nab-paclitaxel plus gemcitabine versus mFOLFIRINOX in the first-line treatment of metastatic pancreatic cancer: a retrospective study

**DOI:** 10.1186/s12957-023-02896-z

**Published:** 2023-01-23

**Authors:** Lei Yang, Jing Su, Wenbo Wang, Fuxiang Zhou

**Affiliations:** 1 Hubei Cancer Clinical Study Center, Hubei Key Laboratory of Tumor Biological Behaviors, Zhongnan Hospital, Wuhan University, Wuhan, China; 2 Department of Radiation Oncology & Medical Oncology, Zhongnan Hospital, Wuhan University, No 169 Donghu Road, Wuchang District, Hubei, Wuhan 430071 China

**Keywords:** Metastatic pancreatic cancer, Chemotherapy, Prognostic nutrition index, Prognosis

## Abstract

**Background:**

Nab-paclitaxel plus gemcitabine (AG) and modified FOLFIRINOX (FFX) are two systemic therapies that have been widely used as standard first-line chemotherapy regimens in metastatic pancreatic cancer. However, since there is no clinical trial to directly compare the efficacy and safety of the two regimens, it is not clear which regimen is more effective. In this study, we aim to examine and compare the efficacy and safety of AG and FFX as first-line chemotherapy regimens in Chinese patients with metastatic pancreatic cancer in a real-world setting.

**Methods:**

We retrospectively evaluated the outcomes of 44 patients who were diagnosed with metastatic pancreatic cancer and were treated with either AG (*n* = 24) or FFX (*n* = 20) as first-line chemotherapy between March 2017 and February 2022 at Zhongnan Hospital of Wuhan University. Prognostic nutrition index (PNI) was calculated based on the serum albumin level and peripheral lymphocyte count. According to the optimal cutoff value of PNI, patients were divided into low PNI group (PNI < 43.70) and high PNI group (PNI ≥ 43.70).

**Results:**

Of 44 patients in this study, 24 were treated with AG, and 20 were treated with FFX as first-line chemotherapy. No significant differences in baseline characteristics were found between the two groups. The objective response rate (ORR) was 16.7% in the AG group and 20.0% in the FFX group. The disease control rate (DCR) was 70.8% in the AG group and 60.0% in the FFX group. There was no significant difference in PFS or OS between the AG group and the FFX group. The median progression-free survival (PFS) was 4.67 months (95% confidence interval [CI], 2.91–6.42) in the AG group and 3.33 months (95% CI, 1.87–4.79, *p* = 0.106) in the FFX group. The median overall survival (OS) was 9.00 months (95% CI, 7.86–12.19) in the AG group and 10.00 months (95% CI, 7.70–12.27, *p* = 0.608) in the FFX group. The second-line treatment rate was 62.5% in the AG group and 55.0% in the FFX group. Immune checkpoint inhibitors (ICIs) based regimens are common second-line treatment options whether in AG or FFX group. Significantly more grade 3–4 peripheral neuropathy occurred in the AG than FFX groups (4 (20.8%) vs 0 (0.0%), *p* = 0.030*). The patients in the PNI (Prognostic nutrition index) ≥ 43.7 group had a significant longer median OS (PNI ≥ 43.7 vs PNI < 43.7: 10.33 vs 8.00 months, *p* = 0.019).

**Conclusion:**

AG and FFX showed comparable efficacy outcomes in patients with metastatic pancreatic cancer. Pancreatic cancer patients receiving first-line chemotherapy with good nutritional status are likely to have a better prognosis.

## Introduction

Pancreatic cancer is one of the most lethal malignancies with very poor prognosis [1, 2]. Moreover, most patients with pancreatic cancer are diagnosed at an advanced stage and have lost the opportunity to receive R0 surgery. With almost as many deaths (*n* = 121,853) as new cases (*n* = 124,994), pancreatic cancer has become the seventh leading cause of cancer-related death in China [3].

Chemotherapy is still the main treatment for metastatic pancreatic cancer. At present, Nab-paclitaxel plus gemcitabine (AG) and modified FOLFIRINOX (a combination regimen consisting of oxaliplatin, irinotecan, 5-fluorouracil, and leucovorin) are generally considered as the standard chemotherapy regimens in metastatic and advanced pancreatic cancer. In 2011, the randomized phase III PRODIGE4/ACCORD11 clinical trial demonstrated that the FOLFIRINOX regimen showed better survival benefits in metastatic pancreatic cancer patients when compared with gemcitabine monotherapy [4]. In 2013, another randomized phase III clinical trial, MPACT, evaluated the efficacy and safety of Nab-paclitaxel plus gemcitabine (AG) regimen in metastatic pancreatic cancer patients. The MPACT trial showed that a combination of gemcitabine and nab-paclitaxel showed significant survival benefits when compared with gemcitabine monotherapy [5]. Thus, based on the above two clinical studies, these two regimens have been recommended as the standard first-line treatment for metastatic pancreatic cancer by various clinical guidelines [6, 7].

Although some studies have compared the efficacy of the two chemotherapy regimens, it remains unclear which is more effective. At present, there is no prospective study comparing AG to FFX directly in metastatic pancreatic cancer. In PRODIGE4/ACCORD11 and MPACT trials, the AG regimen showed numerically worse results than FFX in overall survival (OS) (8.5 *vs* 11.1 months) and progression-free survival (PFS) (5.5 *vs* 6.4 months) [4, 5]. However, in the analysis of these two studies, direct comparison is not convincing due to the different enrolled populations and study designs. Several retrospective studies have found that there is no significant difference between the two chemotherapy regimens [8–10]. Moreover, a network meta-analysis including twenty-two studies in 2021 reported that the survival and toxicity of these two chemotherapy regimens were similar in advanced pancreatic cancer [11]. However, a retrospective study in Korea concluded that FFX would be a better first-line treatment choice than AG as FFX achieved a longer overall survival in metastatic pancreatic cancer [12]. In addition, a retrospective study of advanced pancreatic cancer from Japan found that although no significant differences were found between the efficacy of AG and FFX, the AG regimen had a higher objective response rate and lower toxicity [13]. Therefore, the AG regimen may be more suitable as the first-line treatment for advanced pancreatic cancer. Studies in different countries seem to have different or even opposite results. Therefore, more studies are needed to study the efficacy and safety of these two regimens.

However, even with the progress of medical technology, pancreatic cancer is still resistant to conventional chemotherapy. It is very important to explore the key molecular mechanisms related to the occurrence, development and metastasis of pancreatic cancer. A series of studies have explored the role of long non-coding RNAs [14] and new targeted drugs [15, 16] in pancreatic cancer, which has potential important clinical value.

In this study, we retrospectively reviewed the efficacy and safety of AG and FFX as first-line chemotherapy regimens in metastatic pancreatic cancer patients in our hospital.

## Materials and methods

### Patient eligibility

We retrospectively reviewed the clinical data of patients with metastatic pancreatic cancer who have treated with either AG or FFX regimen as first-line chemotherapy between March 2017 and February 2022 at Zhongnan Hospital of Wuhan University. Eligible patients were as follows: (i) over 18 years old; (ii) histologically diagnosed with pancreatic adenocarcinoma; (iii) had an Eastern Cooperative Oncology Group PS of 0–1; (iv) had at least one measurable lesion measurable disease; (v) and underwent at least two cycles of chemotherapy treatment. This study was conducted following the ethical guidelines of the Helsinki Declaration (revised in 2013). The study was approved by the Ethics Committee of Zhongnan Hospital of Wuhan University (20220127 K), and informed consent was taken from all the patients.

### Treatment and toxicity

Chemotherapy was performed as follows: Nab-paclitaxel (125 mg/m2) followed by gemcitabine (1000 mg/m2) were administered on days 1 and 8 every 3 weeks (AG regimen). FOLFIRINOX regimen was administered in combination with oxaliplatin (85 mg/m^2^), irinotecan (180 mg/m^2^), leucovorin (400 mg/m^2^), and 5-fluorouracil (400 mg/m^2^ bolus, 2400 mg/m^2^ continuous intravenous infusion for 46 h) every 14 days. All patients in this study received chemotherapy treatment until the progression of the disease, unacceptable toxicity, or patient refusal. Dose reduction was evaluated at the discretion of the clinician according to the general condition and toxicities of patients. Treatment-related toxicity was graded according to the National Cancer Institute Common Terminology Criteria for Adverse Events, version 5.0 (CTCAE 5.0).

### Efficacy and survival outcomes

All patients in the present study were followed up until June 30, 2022. According to response evaluation criteria in solid tumors (RECIST version 1.1), tumor response was evaluated by the clinician every 4–8 weeks using enhanced computed tomography and/or magnetic resonance imaging (MRI). The primary outcomes of the analysis included overall survival (OS), progression-free survival (PFS), and objective response rate (ORR).

### Statistical analysis

Statistical analysis in this study was performed using SPSS software version 25 (IBM, NC, USA). Continuous data were expressed as median while categorical data as frequency (percentage). Kaplan–Meier survival curves and the Log-rank test was used to compare the OS and PFS of patients between the two treatment groups. The multivariate Cox regression analysis was used to identify the independent prognostic factors. Statistical significance was determined at *P* < 0.05.

## Results

### Baseline characteristics

The outcomes of 69 patients with metastatic pancreatic cancer were identified retrospectively from March 2017 to February 2022. Of these, 5 patients had no post-baseline assessment, and 10 had no follow-up data, leaving 44 eligible patients (AG group *n* = 24, FFX group *n* = 20).

As shown in Table 1, the baseline characteristics of the 44 patients were summarized. Twenty-four patients were treated with AG (6 females and 18 males, median age 60.0 years) and 20 patients with FFX (7 females and 13 males, median age 55.9 years). All patients had good general conditions (ECOG score of 0 or 1). The liver is the most common site of metastasis in both groups. There was no significant difference in baseline characteristics between the two groups.

**Table 1:** Baseline patients’ characteristics, *n* (%)

Variable	AG (*n* = 24)	FFX (*n* = 20)	*P* value
Age (years)			
Mean ± SD	60.0 ± 9.2	55.9 ± 9.6	0.545
< 60	11 (45.8)	11 (55.0)	
≥ 60	13 (54.2)	9 (45.0)	
Gender			
Male	18 (75.0)	13 (65.0)	0.469
Female	6 (25.0)	7 (35.0)	
Baseline CA19-9, U/mL			
Normal (0–37)	7 (29.2)	6 (30.0)	0.952
Elevated (> 37)	17 (70.8)	14 (70.0)	
Baseline CEA, ng/mL			
Normal (0–7.2)	13 (54.2)	14 (70.0)	0.283
Elevated (> 7.2)	11 (45.8)	6 (30.0)	
Tumor site of pancreas			
Head/neck	13 (54.2)	13 (65.0)	0.467
Body/tail	11 (45.8)	7 (35.0)	
Number of metastasis			
1	11 (45.8)	9 (45.0)	0.956
≥ 2	13 (54.2)	11 (55.0)	
Liver metastasis			
Yes	18 (75.0)	12 (60.0)	0.287
No	6 (25.0)	8 (40.0)	
Combined with radiotherapy			
Yes	9 (37.5)	13 (65.0)	0.069
No	15 (62.5)	7 (35.0)	
PNI (prognostic nutrition index)	43.55 ± 5.12	43.00 ± 5.08	0.635

### Efficacy

The median follow-up time was 11.5 months (range 2.0–22.5 months) as of June 30, 2022. As shown in Table 2, no patient in either treatment group achieved a complete response (CR). In the AG group, 4 patients experienced PR (PR, 16.7%), 13 patients showed stable disease (SD, 54.2%), and 6 patients had progressive disease (PD, 29.2%) according to RECIST version 1.1. In the FFX group, 4 patients experienced partial responses (PR, 20.0%), 8 patients showed stable disease (SD, 40.0%), and 8 patients had (PD, 40.0%) according to RECIST version 1.1. The disease control rates (DCR) were 70.8% and 60.0% in the AG group and FFX group, respectively. However, no statistical difference was found between the two groups in terms of tumor responses (Table 2). As shown in Table 3, in the AG group, 15 (62.5%) of 24 patients received second-line treatment, 9 patients received 5FU-based regimens, and 6 patients received PD-1-based regimens. While in the FFX group, 11 (55.0%) of 20 patients received second-line treatment, and PD-1-based regimens were most commonly used as secondary chemotherapy (*n* = 5, 20.0%).

**Table 2:** The difference of tumor response between two groups, *n* (%)

Tumor response	AG (*n* = 24)	FFX (*n* = 20)	*P* value
Objective response rate(%)	16.7%	20.0%	0.775
Complete response (CR)	0 (0.0)	0 (0.0)	
Partial response (PR)	4 (16.7)	4 (20.0)	
Stable disease (SD)	13 (54.2)	8 (40.0)	
Progressive disease (PD)	7 (29.2)	8 (40.0)	
Disease control rate (PR + SD)	17 (70.8)	11 (60.0)

**Table 3:** Second-line treatment

**Second-line treatment**	AG (*n* = 24), *n* (%)	FFX (*n* = 20), *n* (%)
AG	0	3 (15.0)
FFX	2 (8.3)	0
Oxaliplatin plus S1	3 (12.5)	0
PD-1	3 (12.5)	0
PD-1 + apatinib	2 (8.3)	3 (15.0)
PD-1 + A	1 (4.2)	1 (5.0)
PD-1 + gemcitabine	0	1 (5.0)
S1 or capecitabine	4 (16.7)	1 (5.0)
Aptatinib or lenvatinib	0	1 (5.0)
Gemcitabine plus S1	0	1 (5.0)
**Total**	**15 (62.5%)**	**11 (55.0%)**

### Survival

**Fig. 1:**
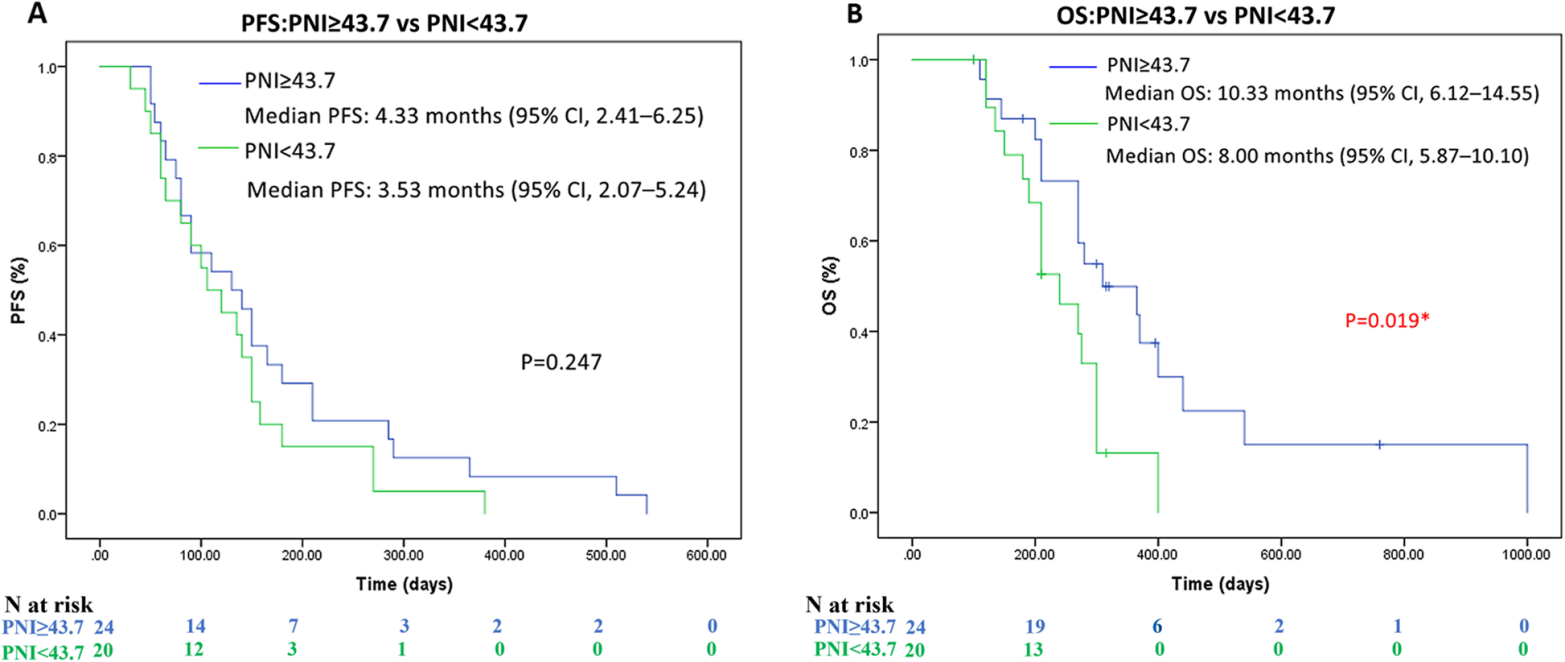
Progression-free survival (**A**) and overall survival (**B**) of each regimen

The Kaplan–Meier curves for PFS and OS were shown in Fig. 1. The median PFS was 4.67 months (95% CI, 2.91–6.42) in the AG group and 3.33 months (95% CI, 1.87–4.79) in the FFX group (*p* = 0.106), respectively (Fig. 1A). The median OS was 9.00 months (95% CI, 7.86–12.19) with the AG group as compared with 10.00 months (95% CI, 7.70–12.27) with the FFX group (*p* = 0.608), respectively (Fig. 1B).

### Subgroup analysis

**Fig. 2:**
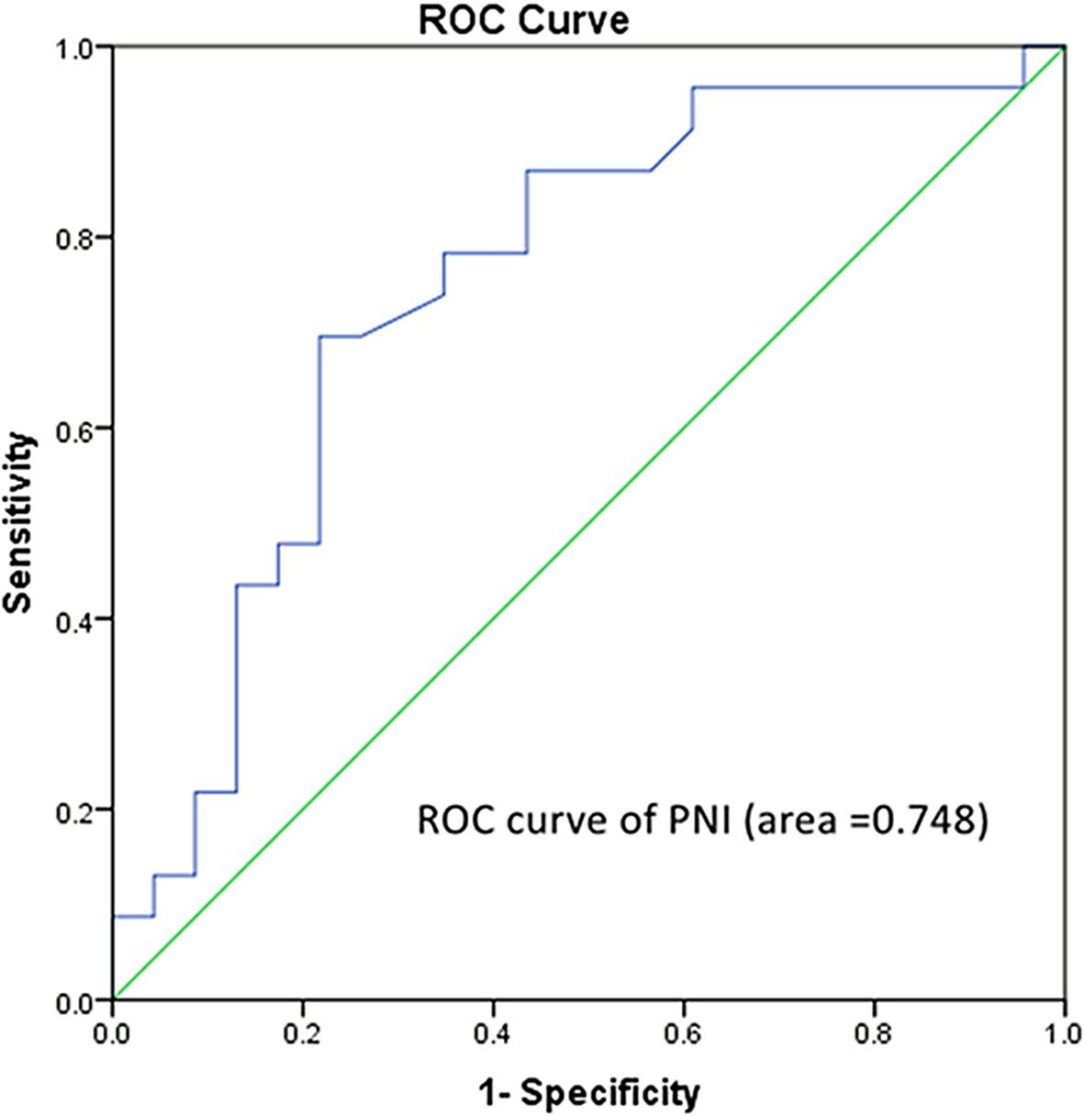
Receiver operating characteristic (ROC) analysis was established to determine the optimal cut-off value of PNI

**Fig. 3:**
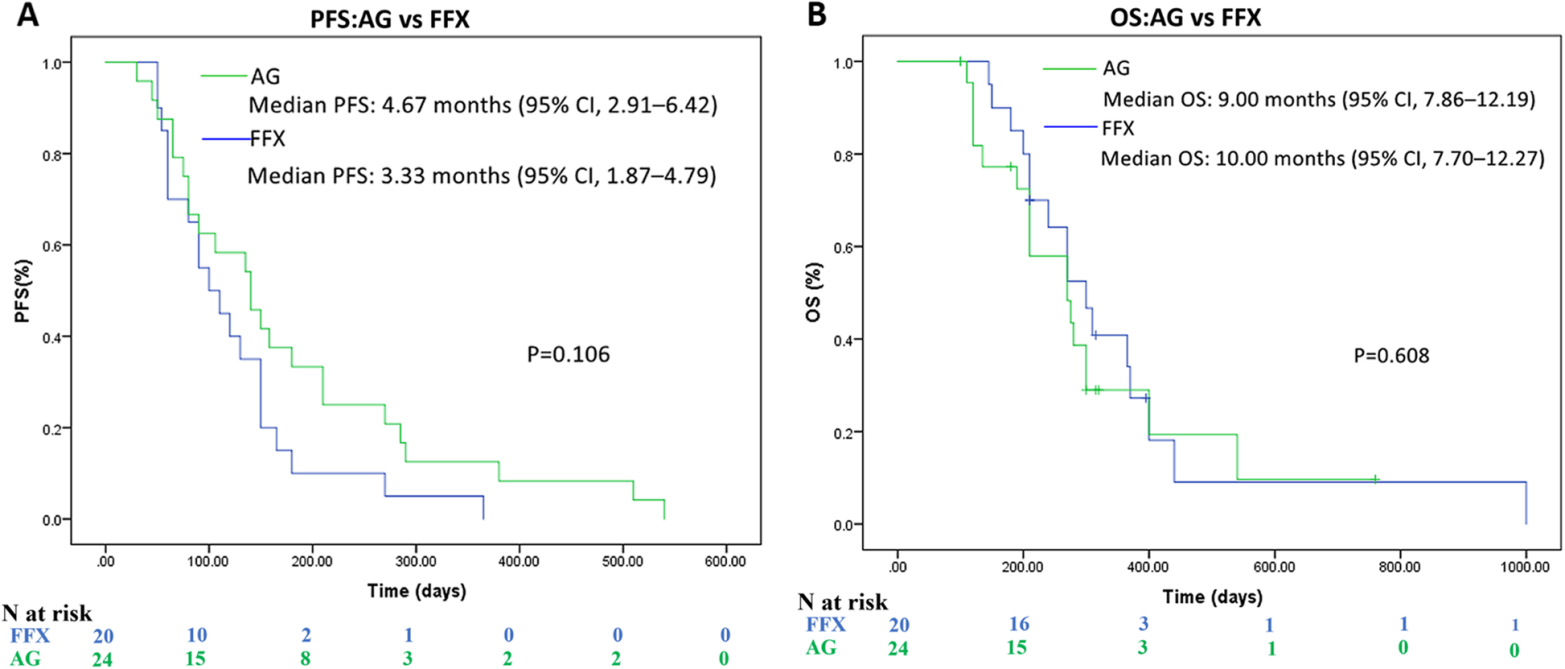
Progression-free survival (**A**) and overall survival (**B**) of whole populations according to different PNI status

PNI was calculated based on the serum albumin level and peripheral lymphocyte count. The median PNI was 43.50 in this study. As shown in Fig. 2, receiver operating characteristic (ROC) analysis was established to determine the optimal cut-off value of PNI. In this study, the optimal cut-off value of PNI is 43.70 (sensitivity, 73.70%; specificity 78.30%). The area under curve (AUC) is 0.748 (95% CI = 0.601 ~ 0.894, *p* = 0.004). Then, patients were divided into low PNI group (PNI < 43.70) and high PNI group (PNI ≥ 43.70). Subgroup analyses of overall survival according to stratification factors showed that PNI was significantly related to the OS (Fig. 3). When comparing PNI ≥ 43.70 and PNI < 43.70, the patients in the two groups showed significant differences in OS. The patients in the high PNI group had a significant longer median OS (PNI ≥ 43.7 vs PNI < 43.7: 10.33 vs 8.00 months, *p* = 0.019).

Compared with low PNI group, high PNI group had a numerically longer PFS (PNI ≥ 43.7 vs PNI < 43.7: 4.33 vs 3.53 months), but no statistical difference was found (*p* = 0.247).

### Univariate and multivariate analysis

Univariate and multivariable analyses are shown in Table 4. In univariate analysis, PNI (prognostic nutrition index) was significantly associated with OS (*p* < 0.05). Similarly, multivariate analysis revealed that a low PNI level (HR = 2.252 [95% CI, 1.021–4.959]; *p* = 0.044) was an independent prognostic factor for worse OS.

**Table 4:** Univariable and multivariable analysis for overall survival (OS)

Variables	Univariable analysis		Multivariable analysis	
	**HR (95% CI)**	***P* value**	**HR (95% CI)**	***P* value**
Treatment regimen				
FFX vs AG	1.193 (0.589–2.523)	0.621		
Age (years)				
≥ 60 vs < 60	0.742 (0.365–1.511)	0.411		
Gender				
Male vs female	0.999 (0.465–2.146)	0.997		
Baseline CA19-9, U/mL				
Elevated (> 37) vs normal	0.976 (0.449–2.121)	0.951		
Baseline CEA, ng/mL				
Elevated (> 7.2) vs normal	0.731 (0.363–1.473)	0.381		
Tumor site of pancreas				
Head/neck vs body/tail	0.724 (0.354–1.479)	0.375		
Number of metastasis				
≥ 2 vs 1	1.672 (0.818–3.416)	0.158		
Liver metastasis				
Yes vs no	1.857 (0.844–4.085)	0.124		
Combined with radiotherapy				
Yes vs no	0.697 (0.347–1.401)	0.311		
PNI (prognostic nutrition index)				
< 43.7 vs ≥ 43.7	2.310 (1.098–4.858)	0.027*	2.252 (1.021–4.959)	0.044*

### Adverse events

As shown in Table 5, the most frequent grade 3/4 toxicity was neutropenia. The grade 3/4 neutropenia rate was both 25.0% in the two groups. One patient in each group developed febrile neutropenia (FN). Moreover, significantly more grade 3/4 peripheral neuropathy occurred in the AG group (AG vs FFX: 5 (20.8%) vs 0 (0.0%), *p* = 0.030). No drug-related death in either group occurred.

**Table 5:** Grade 3–4 adverse events occurring in patients

Grade 3–4 adverse events	AG (*n* = 24), *n* (%)	FFX (*n* = 20), *n* (%)	*P* value
Peripheral neuropathy	5	0	0.030*
Neutropenia	6	5	0.676
Febrile neutropenia	1	1	0.895
Thrombocytopenia	5	2	0.328
Vomiting	1	3	0.213
Fatigue	2	0	0.552
Diarrhea	2	2	0.848

## Discussion

Based on the results of the PRODIGE4/ACCORD11 and MPACT clinical trials, modified FOLFIRINOX (FFX) and Nab-paclitaxel plus Gemcitabine (AG) are frequently recommended as first-line treatment regimens for metastatic pancreatic cancer [4, 5]. Some retrospective or real-world studies have compared the effectiveness and safety of the two regimens [8–10, 12, 13]. However, since there is no clinical trial to directly compare the efficacy and safety of the two regimens, it is not clear which regimen is more effective. Additionally, the majority of these studies included not only metastatic pancreatic cancer (mPC) but also local advanced pancreatic cancer (LAPC) or borderline resectable tumors, resulting in a very heterogeneous study.

In this retrospective study, we aimed to compare the two standard first-line chemotherapy regimens only in Chinese patients with metastatic pancreatic cancer. Herein, our study showed no difference in terms of survival outcomes or tumor response between the patients treated with AG or FFX. Moreover, high PNI was a good prognostic factor for OS in the whole patients included in this study.

Previously, a randomized phase III trial showed that gemcitabine significantly improved disease-related symptoms and median OS compared with 5-fluorouracil alone in patients with locally advanced or metastatic pancreatic cancer [17]. Thus, gemcitabine monotherapy became the standard chemotherapy regimen for advanced pancreatic cancer since the 1990s. However, since more effective alternatives regimens have been developed, including AG or FFX, recent guidelines recommend the use of AG or FFX regimens for first-line treatment in locally advanced or metastatic pancreatic cancer based on the results of the MPACT trial and PRODIGE4/ACCORD11 trials [6, 7].

The present study showed that the overall survival of the AG group and FFX group as first-line therapy in metastatic pancreatic cancer were 9.00 months and 10.00 months respectively, consistent with their respective pivotal trials [4, 5]. The objective response rates (ORR) were 16.7% and 20.0% in the AG group and FFX group, respectively. No statistical difference was found between the two groups in terms of tumor responses. Immune checkpoint inhibitors (ICIs) based regimens are common second-line treatment options whether in AG or FFX group. Immunotherapy has reshaped the therapeutic pattern of many solid tumors, including lung cancer, gastric cancer, and melanoma [18–21]. Moreover, various clinical trials have also begun to explore the effect of immunotherapy on pancreatic cancer. An open-label, single-center, phase Ib/II clinical trial, which used toripalimab (anti-PD-1) plus AG as the first-line treatment for patients with locally advanced or metastatic pancreatic cancer, showed a favorable response and manageable toxicity [22]. Another randomized phase 2 trial conducted by researchers at the university of Pennsylvania and parker institute for cancer immunotherapy evaluated the efficacy of nivolumab (anti-PD-1) and/or sotigalimab (CD40 agonistic antibody) with AG in patients with first-line metastatic pancreatic cancer [23]. Thus, immunotherapy combined with chemotherapy may be a better treatment for advanced pancreatic cancer, which is worth further exploration and research.

The present study also found that PNI (prognostic nutritional index) was significantly related to the OS in metastatic pancreatic cancer. The patients in the high PNI group had a significantly longer median OS in this study. Nutritional status and immunity are closely related to the prognosis of patients with malignant tumors, according to many studies [24–28]. PNI is an easily accessible index that considers nutritional and immunologic factors. The preoperative nutritional index was first proposed by Buzby et al. [29] in 1980 and was initially used to assess preoperative nutritional status, surgical risk, and postoperative complications (PNI = 5 × total lymphocyte count (10^9^/L) + serum albumin (g/L)). According to recent studies, PNI can be used to evaluate the prognosis of many cancers. Our study reported that low PNI was significantly associated with reduced survival in patients with metastatic pancreatic cancer. Additionally, studies have shown that nutritional interventions can reduce the risk of death among cancer patients. A phase III, randomized, controlled trial showed that early nutrition and psychological intervention could reduce the mortality risk of patients with advanced esophageal cancer by 32% [30]. A prospective, multicenter, randomized study showed that whole-course nutrition management is helpful to maintain the weight and nutritional status of esophageal cancer patients receiving concurrent radiotherapy and chemotherapy and improving their treatment tolerance and short-term prognosis [31]. Nutritional support may improve the prognosis of pancreatic cancer, which deserves further attention and research.

Non-systematic treatment of advanced and metastatic pancreatic cancer also has important clinical value. Due to the local expansion of pancreatic cancer, many pancreatic cancer patients may experience severe abdominal pain, which may seriously affect the quality of life of patients. Endoscopic ultrasonography guided-celiac plexus neurolysis (EUS-CPN) is an important option for the treatment of severe intractable pain in pancreatic cancer patients. Moreover, a retrospective study found that EUS-guided tumor ablation combined with CPN may be more effective and safe compared to standard EUS-CPN [32]. Meanwhile, radiotherapy is also widely used to relieve pain of pancreatic cancer [33].

This study has limitations. This study was a single-center retrospective study involving a relatively small number of patients with pancreatic cancer. The results need to be further confirmed by a large sample prospective study.

In conclusion, in this retrospective study, we aimed to compare the two standard first-line chemotherapy regimens only in Chinese patients with metastatic pancreatic cancer. Herein, our study showed no significant difference in terms of survival outcomes or tumor response between the patients treated with AG or FFX. Moreover, high PNI was a good prognostic factor for OS in the whole patients included in this study. Further studies are needed to prove which regimen works better in a wider range of situations. However, consistent with other research results, our study also suggested that the prognosis of patients with metastatic pancreatic cancer was very poor. Therefore, chemotherapy combined with targeted therapy or immunotherapy is worthy of further research as a more effective approach.

## Data Availability

The data that support the findings of this study are available from the corresponding author upon reasonable request.

## References

[1] Park W, Chawla A, O'Reilly EM (2021). Pancreatic cancer: a review. JAMA.

[2] Siegel RL, Miller KD, Fuchs HE, Jemal A (2021). Cancer Statistics, 2021. CA Cancer J Clin.

[3] Zheng R, Zhang S, Zeng H (2022). Cancer incidence and mortality in China, 2016. J Natl Cancer Center.

[4] Conroy T, Desseigne F, Ychou M (2011). Groupe Tumeurs Digestives of Unicancer; PRODIGE Intergroup. FOLFIRINOX versus gemcitabine for metastatic pancreatic cancer. N Engl J Med..

[5] Von Hoff DD, Ervin T, Arena FP (2013). Increased survival in pancreatic cancer with nab-paclitaxel plus gemcitabine. N Engl J Med.

[6] Tempero MA, Malafa MP, Chiorean EG (2019). Pancreatic adenocarcinoma, Version 1.2019. J Natl Compr Canc Netw.

[7] Sohal DP, Mangu PB, Khorana AA (2016). Metastatic pancreatic cancer: American Society of Clinical Oncology Clinical Practice Guideline. J Clin Oncol.

[8] Williet N, Petrillo A, Roth G (2021). Gemcitabine/Nab-Paclitaxel versus FOLFIRINOX in locally advanced pancreatic cancer: a European multicenter study. Cancers (Basel).

[9] Riedl JM, Posch F, Horvath L (2021). Gemcitabine/nab-paclitaxel versus FOLFIRINOX for palliative first-line treatment of advanced pancreatic cancer: a propensity score analysis. Eur J Cancer.

[10] Lee JC, Woo SM, Shin DW (2020). Comparison of FOLFIRINOX and gemcitabine Plus Nab-paclitaxel for treatment of metastatic pancreatic cancer: using Korean Pancreatic Cancer (K-PaC) Registry. Am J Clin Oncol.

[11] Chen J, Hua Q, Wang H (2021). Meta-analysis and indirect treatment comparison of modified FOLFIRINOX and gemcitabine plus nab-paclitaxel as first-line chemotherapy in advanced pancreatic cancer. BMC Cancer.

[12] Chun JW, Lee SH, Kim JS (2021). Comparison between FOLFIRINOX and gemcitabine plus nab-paclitaxel including sequential treatment for metastatic pancreatic cancer: a propensity score matching approach. BMC Cancer.

[13] Tahara J, Shimizu K, Otsuka N (2018). Gemcitabine plus nab-paclitaxel vs. FOLFIRINOX for patients with advanced pancreatic cancer. Cancer Chemother Pharmacol.

[14] Kirtonia A, Pandey AK, Ramachandran B (2022). Overexpression of laminin-5 gamma-2 promotes tumorigenesis of pancreatic ductal adenocarcinoma through EGFR/ERK1/2/AKT/mTOR cascade. Cell Mol Life Sci.

[15] Pandya G, Kirtonia A, Sethi G (2020). The implication of long non-coding RNAs in the diagnosis, pathogenesis and drug resistance of pancreatic ductal adenocarcinoma and their possible therapeutic potential. Biochim Biophys Acta Rev Cancer..

[16] Chien W, Sudo M, Ding LW (2018). Functional genome-wide screening identifies targets and pathways sensitizing pancreatic cancer cells to dasatinib. J Cancer.

[17] Burris HA, Moore MJ, Andersen J (1997). Improvements in survival and clinical benefit with gemcitabine as first-line therapy for patients with advanced pancreas cancer: a randomized trial. J Clin Oncol.

[18] Ai L, Chen J, Yan H (2020). Research status and outlook of PD-1/PD-L1 inhibitors for cancer therapy. Drug Des Devel Ther.

[19] Ren S, Xiong X, You H (2021). The combination of immune checkpoint blockade and angiogenesis inhibitors in the treatment of advanced non-small cell lung cancer. Front Immunol..

[20] Joshi SS, Badgwell BD (2021). Current treatment and recent progress in gastric cancer. CA Cancer J Clin.

[21] Carlino MS, Larkin J, Long GV (2021). Immune checkpoint inhibitors in melanoma. Lancet.

[22] Shui L, Cheng K, Li X (2020). Study protocol for an open-label, single-arm, phase Ib/II study of combination of toripalimab, nab-paclitaxel, and gemcitabine as the first-line treatment for patients with unresectable pancreatic ductal adenocarcinoma. BMC Cancer.

[23] Padrón LJ, Maurer DM, O'Hara MH (2022). Sotigalimab and/or nivolumab with chemotherapy in first-line metastatic pancreatic cancer: clinical and immunologic analyses from the randomized phase 2 PRINCE trial. Nat Med.

[24] Li Y, Wang WB, Yang L (2022). The combination of body composition conditions and systemic inflammatory markers has prognostic value for patients with gastric cancer treated with adjuvant chemoradiotherapy. Nutrition..

[25] Kubo Y, Tanaka K, Yamasaki M (2021). Influences of the Charlson Comorbidity Index and nutrition status on prognosis after esophageal cancer surgery. Ann Surg Oncol.

[26] Guo ZQ, Yu JM, Li W (2020). Investigation on the Nutrition Status and Clinical Outcome of Common Cancers (INSCOC) Group. Survey and analysis of the nutritional status in hospitalized patients with malignant gastric tumors and its influence on the quality of life. Support Care Cancer..

[27] Yamamoto T, Kawada K, Obama K (2021). Inflammation-related biomarkers for the prediction of prognosis in colorectal cancer patients. Int J Mol Sci.

[28] Deftereos I, Kiss N, Isenring E (2020). A systematic review of the effect of preoperative nutrition support on nutritional status and treatment outcomes in upper gastrointestinal cancer resection. Eur J Surg Oncol.

[29] Buzby GP, Mullen JL, Matthews DC (1980). Prognostic nutritional index in gastrointestinal surgery. Am J Surg.

[30] Lu Z, Fang Y, Liu C (2021). Early Interdisciplinary supportive care in patients with previously untreated metastatic esophagogastric cancer: a phase III randomized controlled trial. J Clin Oncol.

[31] Lyu J, Shi A, Li T (2022). Effects of enteral nutrition on patients with oesophageal carcinoma treated with concurrent chemoradiotherapy: a prospective, multicentre, randomised, controlled study. Front Oncol..

[32] Facciorusso A, Di Maso M, Serviddio G (2017). Echoendoscopic ethanol ablation of tumor combined with celiac plexus neurolysis in patients with pancreatic adenocarcinoma. J Gastroenterol Hepatol.

[33] Wolny-Rokicka E, Sutkowski K, Grządziel A (2016). Tolerance and efficacy of palliative radiotherapy for advanced pancreatic cancer: a retrospective analysis of single-institutional experiences. Mol Clin Oncol.

